# Dostarlimab as a Miracle Drug: Rising Hope against Cancer Treatment

**DOI:** 10.3390/bios12080617

**Published:** 2022-08-08

**Authors:** Vanshikha Singh, Afsana Sheikh, Mohammed A. S. Abourehab, Prashant Kesharwani

**Affiliations:** 1 Department of Pharmaceutics, School of Pharmaceutical Education and Research, Jamia Hamdard, New Delhi 110062, India; 2 Department of Pharmaceutics, College of Pharmacy, Umm Al-Qura University, Makkah 21955, Saudi Arabia; 3 Department of Pharmaceutics and Industrial Pharmacy, College of Pharmacy, Minia University, Minia 61519, Egypt

**Keywords:** immunotherapy, clinical trial, dostarlimab, colon cancer, cancer therapy, drug delivery

## Abstract

Immunotherapy is one of the four pillars of cancer treatment that has recently emerged as a beacon of hope for cancer patients. Certain immunotherapies, for example, immune checkpoint inhibitor therapy, monoclonal antibody therapy and chimeric antigen T-cell therapy have garnered extensive interest in response to their exceptional properties that activate the immune system to respond to cancer cells, inhibiting their progression. In the era of rapid development, dostarlimab, an anti-programmed cell death protein (PD-1) monoclonal antibody has mesmerized the medical profession by showing complete (100%) cure of patients with colorectal cancer. Not only this, the results obtained from clinical trials revealed no major side effects in any of the participants in the study. Dostarlimab has also shown promising results in endometrial cancer, ovarian cancer, melanoma, head and neck cancer, and breast cancer therapy. This review focuses upon the action of immunotherapy, extensively emphasizing the miraculous therapy to activate T-cells for cancer treatment. Based on this, we discuss major ongoing clinical trials and combination immunotherapies to enlighten future clinicians and researchers about the response of dostarlimab against various cancers.

## 1. Introduction

Cancer remains one of the deadliest diseases that humankind has ever encountered, and despite of years of research in this field it is still a leading health problem responsible for over 10 million deaths per year [1]. Several forms of treatment have been brought to use, including treatment with drugs in chemotherapy, radiation in radiotherapy, surgery, and immunotherapy. Immuno-oncology is the newest field of research in this area and its scope and full potential are yet to be explored. As part of immunotherapy, specific parts of the patient’s immune system are used to treat a range of diseases, including cancer and mostly solid tumors [2,3,4,5,6,7,8]. Cancer immunotherapy aims to re-activate the immune system, which has been suppressed by tumor cells in numerous ways. Several novel strategies involving immunotherapy are being developed to treat cancer or to minimize the associated cytotoxic effects caused as a result of different cancer therapies. Immunotherapies are very specific and when stimulated; they target cancerous stem cell and even metastatic cancer, which in turn highlights their potential of reaching the smallest of tumors where surgeons might not. Immunotherapy has also brought into focus the development of cancer vaccines, which have shown potential results in minimizing tumor growth, but still fall short in eradicating it completely. Additionally, there are several antibody-based drugs employed for cancer treatment which indirectly or directly relate to immunotherapy [9,10,11,12].

Another brilliant insight in cancer immunotherapy was brought forth by the Nobel-prize-winning discovery of T-cell checkpoints such as CTLA-4 and PD1 by Drs. Allison and Honjo [13]. This research highlighted the complexity of immune surveillance and how it can be utilized for building auto immunity. The signals in our body are hardwired to maintain immunity by either fighting against foreign pathogens or different abnormal cells. Restricting T-cell surface receptors by blocking them not only enhances immunity by increasing the immune response against tumors, but also induces certain autoimmune responses. As a form of immunotherapy, adoptive cell therapy (ACT) involves supplying immunologically active cells to a patient for treatment and the prevention of disease formation [14,15]. There are, in total, five classes of immunotherapy, including: checkpoint inhibitors, antibody-based targeted therapies, cancer vaccines, antigenic receptor T-cells, and lastly oncolytic viruses [16,17,18,19]. Today, mAB-based immunotherapy is considered an important part of cancer treatment, alongside other methods. These antibodies can not only target tumor cells, but can also trigger long-lasting antitumor immune responses. The versatility of antibodies as a therapeutic platform has led to the development of new cancer treatment strategies that will change the way cancer is treated in the future.

## 2. Monoclonal Antibody-Based Cancer Immunotherapy

Antibodies are large glycoproteins belonging to the immunoglobulin (Ig) superfamily which are found naturally in blood and are responsible for recognizing foreign antigens, neutralizing them, and evoking further immune responses. They structurally comprise two heavy and two light chains in the shape of a Y. There are five different types of immunoglobins based on the type of heavy chains. These include IgA, IgD, IgE, IgG, and IgM. The Y-shaped immunoglobins consists of different parts, while the Fab (fragment antigen-binding) portion of the antibody is located at each tip of the Y; the fragment crystallizable (Fc) region is present at the base of the Y structure. Antibodies recognize specific antigens by their Fab portion, whereas Fc receptors mediate interactions between antibodies and other components of the immune system. The most common form of immunoglobulin used for antibody-based immunotherapy is IgG, attributed to its interaction with FcR and FcγR, which are largely found in natural killer cells, macrophages, monocytes and granulocytes such as eosinophils or basophils. They are involved in specific functions related to complement-dependent cytotoxicity (CDC) and antibody-dependent cellular cytotoxicity (ADCC). Moreover, IgGs are further subdivided based on the ability of FcR to elicit CDC or ADCC response; while IgG1 and IgG3 can successfully induce these responses, IgG2 and IgG4 cannot [20,21].

mABs means “all for just one” specific type of antibody; that is, each mAB has multiple copies of just one type of antibody isotype meant for targeting a unique antigen. There are various mABs available for the treatment of cancer, and while they all act through different pathways, some of them might act by more than a single process. mAB therapy for cancer has advanced considerably after unmodified murine mAbs were first considered as anticancer agents. Several mAB-based strategies have proven to have good potential in treating cancer patients. Their action involves the use of unlabeled IgG that specifically binds to the tumor cells, or alters the active host response towards the tumor [22]. These mABs are also capable of acting as immunoconjugates to deliver cytotoxic moieties at cancerous sites, altering the specificity of mABs to retarget cellular immunity. The mechanisms involved in the process include: (a) ADCC, which is composed of targeted mABs formed from chimeric or human antibody components that are meant for binding tumor-associated antigens; (b) CDC, as the name suggests, depicts complement activation; (c) and factor and receptor inhibition, which involves restricting the receptors that are involved in the activation of signal pathways for cancer cell proliferation or in the process of angiogenesis. Certain examples of mABs that act by ADCC include transtuzumab, pertuzumab, rituximab and cetuximab, while those acting by CDC are alemtuzumab, ofatumumab, rituximab and cetuximab. mABs used in immunotherapy that bind to a specific antigen are divided into two classes, depending on if they carry any chemotherapeutic drug or radioactive substance. Nonconjugated, self-acting mABs include alemtuzumab and transtuzumab, while the second class of mABs are conjugated with drugs or radioactive substances to deliver these mABs at the cancerous site. Examples of mAB conjugated with drugs include gemtuzumab ozogamicin, whereas ibritumomab thiuxetan is a radioactive, conjugated mAB. It can be seen that understanding the mechanisms by which mABs lyse tumors is vital to achieving more effective treatment. Despite the fact that mABs have different mechanisms of action, all of these have directly or indirectly become a part of the standard treatment protocol in combination with chemotherapy and/or radiation. Indeed, cancer mAb-based therapy investment and the speed of progress are at an all-time high in both the public and private sectors [23].

It is a time of unprecedented progress in mAb-based therapy, with new therapeutic agents and constructs being developed rapidly, with an enhanced understanding of their biological effects and growing clinical experience based on both clinical trials and the community use of FDA-approved products. Dedicated research in the translational and clinical field of mABs have brought forth miraculous results for the very first time in anticancer therapy. A trial performed for dostarlimab on 12 patients with colorectal cancer produced complete cancer recovery. Although, this trial is at the phase II stage and was conducted only on limited individuals, a proven complete cure for cancer makes the world wonder upon this drug with high hope [24,25]. On top of these positive results, another main characteristic feature of the trial was that the drug was not accompanied with any surgery, chemotherapy or radiotherapy. This review provides an overview of dostarlimab, its mechanism of action and information about other checkpoint inhibitor mABs. It also consolidates different ongoing trials of dostarlimab as a monotherapy and as a combination therapy for different cancers including endometrial, ovarian, colorectal, lung, head and neck squamous cell cancer, and many more.

## 3. Dostarilimab and Mechanism of Action

**Figure 1:**
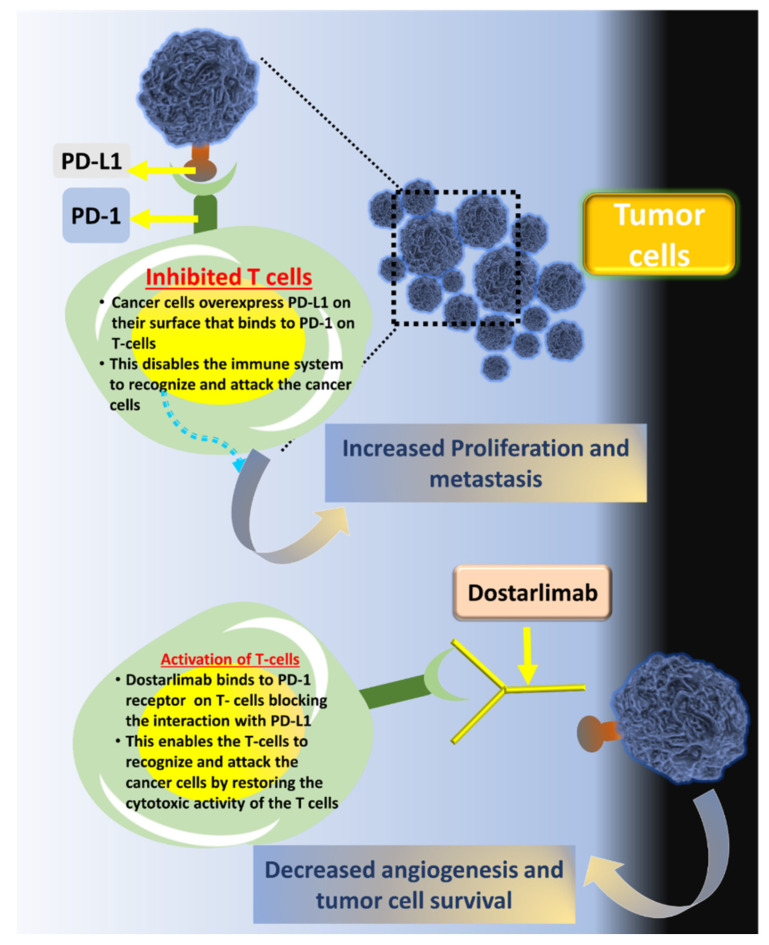
Illustration of the activity of dostarlimab against cancer cells. The PD-1 inhibitor (dostarlimab) inhibits the interaction of T-cells over-expressing PD-1 protein with the ligands (PD-L1) present in cancer cells.

Dostarlimab (Jemperli™) or dostarlimab-gxly is a humanized mAB which acts as an antagonist for programmed death-1 (PD-1) receptors. It is being developed by GlaxoSmithKline (GSK) under a license from AnaptysBio Inc for the treatment of several forms of cancer including endometrial cancer, colorectal cancer, ovarian cancer, cancer of the head and neck, small cell lung cancer (SCLC), non-small cell lung cancer (NSCLC), squamous cell cancer (SCC), fallopian tube cancer, pancreatic cancer, and many more. According to preliminary findings from the GARNET trial, dostarlimab has recently been approved (22 April 2021) for adults with advanced or recurrent advanced mismatch repair-deficient endometrial cancer (dMMR) in the EU and USA. The dose of dostarlimab that is generally recommended is 500 mg every 3 weeks (for the first four doses), after the fourth dose, 1000 mg every 6 weeks is administered until disease progression or any unacceptable toxicity is noticed [26,27,28] (Figure 1).

PD1 is an immune checkpoint receptor found in T-cells that suppresses cancer-specific immune responses. The humanized IgG4 mAB, dostarlimab, is derived from a Chinese hamster ovary cell and has a molecular weight of approx 144 kDa. A binding between the PD-1 ligands (PD-L1 and PD-L2) and the PD-1 receptor on T-cells inhibits cytokine and T-cell proliferation. In some tumors, PD-1 ligands are upregulated, and signaling through this pathway may contribute to the suppression of active T-cell immunity. This is where the drug dostarlimab comes into the picture. It inhibits programmed cell death receptor-1 (PD-1) and blocks the interaction of receptors with PD-L1 and PD-L2, which in turn activates T-cells and enhances overall immunity. Studies have depicted that dostarlimab binds with PD-1 receptors of both humans and cynomolgus monkeys with high affinity, as seen from the results obtained in flow cytometry and plasmon resonance. Moreover, a human CD4+ mixed lymphocyte reaction assay showed that dostarlimab worked as a functional antagonist, resulting in increased IL-2 production. This assay also showed the enhanced activity of dostarlimab when TIM3 antibodies or LAG3 antibodies were present. In the presence of antibodies, dostarlimab exhibited increased activity, but no significant cytokine release was observed from human PBMCs (peripheral blood mononuclear cells) [29].

A pharmacokinetic study for dostarlimab-gxly was performed on patients with solid tumors which included 150 endometrial cancer patients. It was noted that there was a proportionate increase in mean Cmax, AUC0−inf and AUC0−τ over the dose range of 1.0–10 mg/kg. Moreover, the mean cycles of Cmax and AUC0−τ after the administration of 500 mg dostarlimab once every 3 weeks was reported to be in the range of 171 µg/mL and 35,730 µg∙h/mL, respectively, and 309 µg/mL and 95,820 µg∙h/mL, respectively, at a dose of 1000 mg administered once every 6 weeks. Similarly, the study also evidenced the mean steady-state volume of the distribution of dostarlimab to be around 5.3 L, and the mean steady-state clearance to be in the range of 0.007 L/h. There were no clinically significant differences observed in the pharmacokinetics characteristics of dostarlimab based on gender, age, ethnicity, tumor type, or renal or hepatic impairment. Although, there are no studies conducted to determine whether dostarlimab-gxly is carcinogenic or genotoxic. Fertility studies have been performed for this drug on monkeys which, after repeating doses for one and three months, found no significant effects on male or female reproductive organs, although most animals in these studies were not sexually mature by the time of study [30].

The first-in-human study, 4010-01-001, otherwise known as the GARNET trial (NCT02715284), evaluated dostarlimab pharmacokinetics (PK), pharmacodynamics (PD), tolerability, clinical activity and safety across multiple solid cancer types, which included endometrial, NSCL and cancer of the ovaries and fallopian tubes. A modified 3 + 3 design was used to evaluate three weight-based doses (1, 3 and 10 mg/kg) administered every 2 weeks intravenously in Part 1. Part 2A used two fixed-dose regimens, 500 mg every 3 weeks intravenously, and 1000 mg every 6 weeks intravenously was administered in in Part 2B. Data from Part 1 demonstrated a maximum receptor occupancy at 2.4 g/mL dostarlimab serum concentrations. Furthermore, a PK model was constructed using the PK data from Part 1 to predict dostarlimab concentrations that would exceed those leading to maximal receptor occupancy at fixed doses. Similarly, Part 2A demonstrated dose-proportional PK and the median serum trough concentrations to be approximately 40 and 50 ng/mL after a single dose of 500 mg and 1000 mg, respectively [31].

## 4. Ongoing Clinical Trials for Dostarlimab

June 2022 saw a revolutionary discovery in the field of cancer treatment. For the very first time in science, a drug under clinical trial showed the complete eradication of a tumor with no reoccurrence. The mAB-based drug dostarlimab was evaluated for safety under efficacy against locally advanced rectal cancer [32].

Primary locally advanced rectal cancer is also known as stage III rectal cancer and is also indicative of resectable tumors with the involvement of lymph nodes. These tumors are characterized for invading and extending close to the mesorectal fascia. These types of colorectal cancer are generally treated with aggressive chemoradiation, short course radiotherapy, and total mesorectal surgery (TME) surgery. The results from this collective therapy are positive, showing excellent survival rates and low reoccurrence. Moreover, in some cases with locally advanced tumors, a complete removal of the tumor is the most preferred and beneficial option for control and survival [33].

As stated above, the standard method of treatment of locally advanced rectal cancer is radiation and neo-adjuvant chemotherapy followed by the surgical removal of the rectum. Additionally, it has also been noted that the cause of some rectal cancers is a lack of mismatch repair. In the context of metastatic disease, mismatch repair-deficient colorectal cancer responds to the programmed death 1 (PD-1) blockade, thus suggesting that a checkpoint blockade may be effective in mismatch repair-deficient patients. Scientists in partnership with GSK initiated a prospective phase 2 study in patients with stage II or III rectal adenocarcinomas who were mismatch repair-deficient. They were administered with single-agent anti-PD-1 mAB dostarlimab every 3 weeks for 6 months. Although this treatment is supposed to be followed with standard surgery and chemoradiotherapy, the patients who depict a clinically complete response following dostarlimab therapy would not undergo chemotherapy, radiotherapy, or surgery. This is also the primary endpoint for the study. Interim results were obtained from the study completed on a total of 12 patients that had successfully completed treatment with dostarlimab and had also undergone a minimum of 6 months’ follow-up. It was evidenced that all 12 patients (100%; 95% confidence interval, 74 to 100) had a complete clinical response and no form of existing tumor, progression and recurrence was noticed in ^18^F-fluorodeoxyglucose–positron-emission tomography, magnetic resonance imaging, biopsy, digital rectal examination, or endoscopic evaluation. Moreover, no adverse events of grade 3 or higher were reported. The study clearly depicted that a single agent PD1 was highly sensitive to mismatch repair-deficient, locally advanced rectal cancer and could bring about positive results; however, a longer follow-up study still needs to be performed to validate this point [34,35].

Dostarlimab was evaluated in a phase 1 nonrandomized clinical trial for patients with deficient mismatch repair endometrial cancer to assess antitumor activity and safety. Part 1 of this ongoing open-label, multicenter single group study began on 7 March 2016, and enrollment for patients with deficient mismatch mutation repair endometrial cancer began on 8 May 2017. Around 104 women with deficient mismatch mutation repair endometrial cancer were enrolled and each patient received intravenous dostarlimab 500 mg every three weeks for four doses, then 1000 mg every six weeks until disease progression, treatment discontinuation or withdrawal occurred. Specifically, the objective of this study was to evaluate the antitumor activity of dostarlimab on recurrent or advanced dMMR (mismatch repair deficiency) endometrial cancer (EC) patients, using the objective response rate (ORR) which was defined by blinded independent central review (BICR) using Response Evaluation Criteria in Solid Tumors (RECIST) guidelines. A similar concept is the duration of response (DOR), defined as the time from the first documented evidence of complete or partial response to the first documented evidence of disease progression or death, whichever occurs first. Following the first dose of dostarlimab administration, radiographic evaluations were performed 12 weeks after the first dose, every 6 weeks (±10 days) until month 12, and then every 12 weeks thereafter.

The results obtained from this analysis on patients with recurrent or advanced dMMR EC who had progressed after platinum-based chemotherapy and dostarlimab monotherapy were associated with an ORR of 42.3% (95% CI, 30.6–54.6%) in almost 30 patients, 29.6% in around 21 patients, and around 12.7% in 9 patients. The responses were durable, and the median DOR was not reached at 11.2 months in the follow-up period. The safety profile depicted by dostarlimab was manageable and comparable to that of other anti-PD-1 antibodies. Additionally, treatment-related adverse events (TRAEs) accounted for less than 2% of patients discontinuing treatment, and there were no treatment-related deaths. To the best of our knowledge, these results are the largest set of data to date on dMMR EC treated with a PD-1 inhibitor [31,36].

Although cross-trial comparisons cannot be performed, it is generally noted that the response rates with anti-PD-1 therapies appear to be more favorable, as evidenced from the ORR range offered by single-agent therapies that ranged from 13.5% (90% CI, 6.5–27.5%) for bevacizumab to 21–27.3% (95% CI, 15–42.8%) for paclitaxel before the introduction of anti-PD-1 therapies. Although the GARNET trial was a single-group study, the antitumor activity observed in patients with dMMR EC was promising, suggesting that dostarlimab might have a role to play in the treatment of patients with dMMR EC. Dostarlimab demonstrated high ORR and a longer duration of response, reflecting its high potential against cancer. Furthermore, one year after inclusion in the GARNET trial, 74% of patients in the dMMR EC population are still alive. In addition to these wide actions of dostarlimab, a unique characteristic of this drug is its dosing regimen. Patients and caregivers both benefit from this unique dosing schedule after 12 weeks of initial treatment with dostarlimab, which may result in less frequent clinic visits and possibly lower healthcare costs. Altogether, the data from the GARNET study has demonstrated durable anticancer action not in only patients with (MMR-proficient) MMRp and dMMR endometrial cancers, but also for non-EC dMMR solid tumors. According to the GARNET trial data, dostarlimab monotherapy was accelerated for approval in the US as a treatment for recurrent/advanced dMMR solid tumors, following the progressive results obtained from prior treatment. Additionally, it has been approved in both Europe (conditional) and the USA (accelerated) for dMMR/MSI-H and dMMR endometrial cancer, respectively, during and after platinum-based chemotherapy [37,38,39]. Another trial similar to it was conducted for evaluating anti-PD-1/PD-L1 axis therapy for patients suffering from inoperable endometrial cancer. This ongoing study is being performed to establish the safety and efficacy of the drug and its generated antitumor immune response [40].

In the past few years, scientists have also tried exploring the potential of dostarlimab against locally advanced cervical cancer (LACC). They hypothesize that the use of dostarlimab as a consolidation therapy following chemotherapy might enhance progression-free survival rate in patients. Based on this rationale, a randomized, phase II, open-label study was set as maintenance therapy for patients with a high risk of LACC. This ongoing study is a randomized study that began in 28 June 2019, and included around 132 participants. Interim data and hence the results for this study are yet to be reported [41,42].

Lung cancer is another cancer that is accountable for the most cancer-related deaths worldwide. Amongst the type of lung cancer, almost 85% belong to the category of non-small cell lung cancer (NSCLC), for which the primary option for treatment is chemotherapy. In recent years, the introduction of immune checkpoint inhibitors has revolutionized the process of cancer treatment.

In a recent trial, the safety and antitumor activity of dostarlimab were studied in a first-in-human, phase 1, multi-center, open-label, two-part study GARNET cohort of 67 patients with recurrent or advanced NSCLC who had previously been treated with platinum-based chemotherapy. While Part 1 of the study was a dose escalation study and involved the evaluation of pharmacodynamics and pharmacokinetic characteristics of the drug at different doses of 1,3 and 10 mg/kg, Part 2, on the other hand, was conducted in two different subparts: Part 2A evaluated the dose safety and Part 2B dealt with evaluating the clinical efficacy of the drug. Immuno-related objective response rate (irORR) and safety were used as the primary endpoint to determine dostarlimab’s antitumor activity in patients with recurrent or advanced NSCLC. An irORR was defined as the proportion of patients achieving immune-related complete response (irCR) or immune-related partial response (irPR) based on the investigators’ assessment per immune-related RECIST (irRECIST). Monotherapy with dostarlimab produced strong antitumor activity and durable responses across all PD-L1 Tumor Proportion Score (TPS) status subgroups. It was noted that, in NSCLC, the safety profile of dostarlimab was acceptable, with low to a manageable toxicity, and was consistent with that of the other agents that block PD-L1. In the entire study, four patients, i.e., almost 6%, discontinued the study due to treatment-related TEAEs (treatment-emergent adverse effects) (TRAEs), and two deaths were caused because of treatment-emergent adverse effects (TEAEs) which were not considered related to treatment with dostarlimab. Furthermore, dostarlimab is currently being studied as a combination regimen for the treatment of NSCLC, as well as other solid tumors, including in the first-line setting [43].

Besides the above-stated studies performed particularly on some specific kinds of cancer, other trials meant for advanced solid tumors are also being performed for dostarlimab. It is being evaluated for safety and efficacy in the phase 1 GARNET study (NCT02715284) in patients with advanced solid tumors. Participants in Cohort F of the GARNET trial had dMMRs or DNA polymerase epsilon (POLE) mutation non-endometrial solid tumors; the majority had GI origins. The patients were administered with 500 mg of dostarlimab for Q3W for four cycles followed by 1000 mg Q6W until discontinuation. Objective response rate (ORR) and duration of response (DOR) were noted by a blinded independent central review per RECIST. The patients receiving ≥1 dose of the drug were included in the safety analysis (around 144 patients), while the ones that had measurable disease at baseline were included as part of the efficacy analysis at the 6-month follow-up (106 dMMR patients). Results from the study showed that amongst 106 patients, around 99, i.e., 93.4% of them, had gastrointestinal tumors. Moreover, the confirmed ORR in dMMR patients was around 38.7% and the complete response rate was approx. 7.5%. The median duration of follow-up was 12.4 months while median DOR was not achieved. In addition, treatment-related adverse events (TRAEs) were also noted in 68.8% of patients, amongst which almost 8.3% of patients were evidenced to experience at least one grade ≥3 TRAE, the most common of which was an increase in lipase in around 1.4% (two patients). Additionally, no deaths were caused as a result of drug administration and only two patients discontinued drug administration because of TRAE. These results obtained from the study show evidence of the potential antitumor activity of dostarlimab against solid tumors. Lastly, the safety profile was also observed for other cohorts in GARNET and the results obtained were very consistent with low to no immune-related TRAEs [44,45,46].

**Figure 2:**
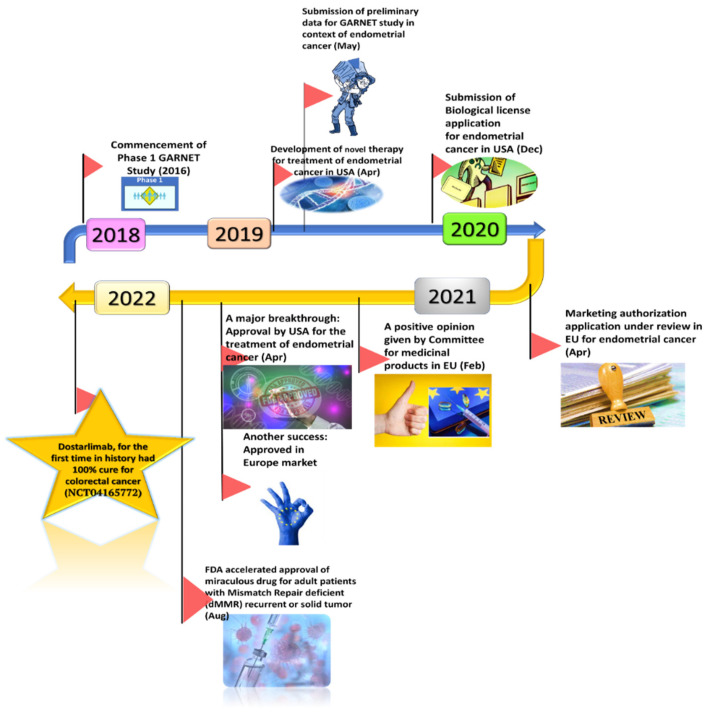
Major milestones of dostarlimab against cancer.

A sarcoma is a malignant solid tumor with high heterogeneity accounting for over 100 subtypes classified so far. Over the years, chemotherapy combined with surgery has proven to be an effective treatment procedure that has resulted in a comparative increase in overall survival rate. Dostarlimab is also being employed to study its activity against sarcomas. A phase II, single-arm, not-randomized, European multicentric study was designed to evaluate the action of TSR-042 (dostarlimab), in patients diagnosed with advanced/metastatic clear cell sarcoma. The study was initiated on February 19, 2021, and around 16 patients were enrolled for the study [47]. The major timestamps of dostarlimab are represented in Figure 2.

## 5. Dostarlimab and Other Combination Therapies under Trial

There are several other immune check point inhibitors such as nivolumab, pembrolizumab, atezolizumab, durvalumab, and avelumab that are utilized for cancer treatment (Table 1). Since dostarlimab is a mAB and not a drug transporter substrate or a cytokine modulator, it is unlikely to show any interactions between other drugs. However, a comparison of dostarlimab based on its pharmacodynamic and pharmacokinetic properties must be conducted with other such immune checkpoint inhibitor-based therapies to obtain a clear and optimal understanding. Dostarlimab, similar to nivolumab and pembrolizumab, specifically targets anti-PD-1 receptors, while atezolizumab, durvalumab and avelumab not only act through anti-PD-1 receptors but also block interaction with the PD-1 and B7.1 receptors. Similarly, the mean peak occupancy for dostarlimab is approx. ~90% and around 85% (70–97%) for nivolumab. The cumulative dose has also been recorded for these drugs: approximately 2-fold for dostarlimab, 3.7-fold for nivolumab, 2.2-fold for pembrolizumab, around 1.91-fold for atezolizumab, and 4.3-fold and 1.25-fold for durvalumab and avelumab, respectively. The three-week dosing schedule is similar for pembrolizumab and nivolumab dose schedules, and ensures the closer monitoring of patients as they begin a new treatment. A dose of 500 mg IV every 3 weeks, then 1000 mg IV every 6 weeks is generally administered as a safety regimen for dostarlimab, while this is 240 mg IV every 2 weeks and 200 mg IV every 3 weeks for nivolumab and pembrolizumab, respectively. Similarly, atezolizumab’s administered dose is 1200 mg or 15 mg/kg IV every 3 weeks; for durvalumab, this is 1500 mg IV every 4 weeks and for ICI avelumab this is 10 mg/kg IV every 2 weeks.

Dostarlimab is also being studied for its activity with one or more chemotherapeutic drugs, including niraparib, pembrolizumab, bevacizumab, cobolimab, and many more. Most of these studies conducted for different cancer types are still under trial. The data below covers some of such ongoing studies.

A combination study investigated the PARPi drug niraparib and anti-PD-1 mAB dostarlimab administered in patients with advanced head and neck squamous cell carcinoma (HNSCC). Niraparib is a type of targeted therapy that inhibits poly adenosine diphosphate-ribose polymerase (PARP), which is an enzyme that repairs DNA in times when it gets damaged. Blocking these PARP might prevent DNA repair in cancerous cells, causing them to die. This phase II trial involving 49 patients was initiated on February 8, 2021 [48]. Researchers hypothesize that combinatory immunotherapy might lead to a reduction in loco-regional recurrence (LRR) and distant metastasis (DM) rates in patients under high risk of HNSCC [49]. Similarly, a phase III trial was designed to study the effect of dostarlimab and niraparib for studying their effects in treating small cell lung cancer and other high-risk neuroendocrine carcinomas. This single-group open-label trial began on February 1, 2021, with an estimated enrollment of 48 patients [50]. Different phase II trials for this combination therapy (dostarlimab and niraparib) are currently being performed for patients with germline or somatic BRCA1/2- and PALB2-mutated pancreatic cancer [51], for breast cancer in patients with BRCA mutations [52], pediatric solid tumors [53], mesothelium NSCLC [54,55], and pancreatic [56], endometrium [57,58] and ovarian cancer [59,60].

In addition to these, other combinations such as cobolimab, docetaxel, and dostarlimab [61]; dostarlimab and pembrolizumab [61]; feladilimab, dostarlimab, and cobolimab [62]; bevacizumab, carboplatin, cobolimab, dostarlimab, niraparib, paclitaxel, and pemetrexed [63]; and dostarlimab, niraparib and pembrolizumab [64] are being studied currently for NSCLC. Moreover, dostarlimab, cobolimab, nivolumab, encelimab, and docetaxel [65]; B intrafusp alfa, cobolimab, dostarlimab, feladilimab, GSK 3174998, and pembrolizumab [66]; and dostarlimab and encelimab [67] are being studied for other colorectal and solid tumors. As interim results from these trials are obtained, more positive points can be reported on behalf of the safety and efficacy of both monotherapy and combination activity.

## 6. Conclusions and Future Prospects

Driving the patient’s own immune system to act against the deadly disease of cancer could potentiate the fast remission of neoplastic cells. Generally, any invasion of pathogens or the non-responsiveness of certain stimuli in the human body turns the T-cell “on”, causing the immune-defense system of the body to respond against them. These T-cells have proteins on their surface called immune checkpoint proteins. Most cancer cells over-express certain proteins that inactivate T-cells, which should be in the field to attack cancer cells in response to their growth and proliferation. Thus, cancer cells switch “off” the immune-response button of T-cells so that they can no longer detect and suppress the cancer cells. Immunotherapy, therefore, acts on tumors, disabling their function to act on T-cells. This, in turn, pushes T-cells to immediately act against them. Dostarlimab is one the immune checkpoint inhibitors that blocks the binding of PD-1 protein on T-cells to the ligand PD-L1/2. It is under trial for different cancer therapies, but, recently, it has shown positive results and complete remission for the very first time in history, and has therefore attracted the interest of clinicians, oncologists, researchers, and even industrialists. The clinical trial was performed on a subset of 12 patients with colorectal cancer with mismatch repair deficiency (MMRd). Such tumors are, however, non-responsive towards radiation or chemotherapy. Nonetheless, in the above trial, all of the 12 patients were completely cured, suggesting that immunotherapy could turn out to be a major milestone in the history of cancer therapy. It is essential to note that all the patients were at same stage of cancer and were given no previous chemotherapy or surgical treatment. The treatment appeared to be effective within this group; however, it is still difficult to suggest that the same response will be reported in large groups of individuals. A phase 3 clinical trial should be carried out covering heterogeneous samples to accurately determine the strength of dostarlimab. Furthermore, studies could be carried out at various locations for different types of cancers as well. As of now, immunotherapy has not reached the wider clinical market and, in this context, the concept of nanotechnology could mark a new beginning in oncotherapy. Overall, it cannot be overlooked that the immunotherapeutic agent dostarlimab is a star compound against colorectal cancer.

## Figures and Tables

**Table 1:** The major clinical trial of dostarlimab.

Drug	Phase of Trial	Cancer Type	Start Date	End Date	No of Participants	Ref.
Dostarlimab	Phase II	Colorectal cancer	11 December 2019	30 November 2025	30	[35]
Dostarlimab	Phase I	Endometrial cancer	15 October 2019	31 October 2024	12	[40]
Dostarlimab	Phase II	Cervical cancer	28 June 2019	December 2024	132	[42]
Dostarlimab	Phase II	Advanced clear cell sarcoma	19 February 2021	1 May 2024	16	[47]
Dostarlimab	Phase II	Endometrial cancer	2 April 2021	February 2023	31	[68]
Dostarlimab	Phase I	Advanced solid tumors	25 June 2020	29 August 2024	178	[69]
Dostarlimab	Phase I	Advanced solid tumors	7 March 2016	30 July 2024	740	[70]
Dostarlimab, niraparib	Phase II	Head and neck squamous cell carcinoma (HNSCC)	8 February 2021	June 2028	49	[48]
Dostarlimab, niraparib	Phase II	Neuroendocrine carcinomas	1 February 2021	30 May 2025	48	[50]
Dostarlimab, niraparib	Phase II	Pancreatic cancer	28 December 2020	1 December 2022	20	[51]
Dostarlimab, niraparib	Phase II	BRCA-mutated breast cancer	18 December 2020	17 July 2029	62	[52]
Dostarlimab, niraparib	Phase I	Pediatric solid tumors	6 October 2020	15 March 2030	116	[53]
Dostarlimab, niraparib	Phase II	Pancreatic cancer	23 July 2020	1 October 2026	25	[56]
Dostarlimab, niraparib	Phase II/III	Endometrial and ovarian cancer	15 July 2020	June 2025	196	[58]
Dostarlimab, niraparib, pembrolizumab	Phase II	SCC, NSCLC	29 September 2017	31 August 2021	53	[64]
Dostarlimab, cobolimab, nivolumab, encelimab, docetaxel	Phase I	Solid tumors	8 July 2016	3 October 2024	369	[65]
Dostarlimab, bintrafusp alfa, cobolimab, feladilimab, GSK 3174998, pembrolizumab	Phase I	Solid tumors	23 June 2016	20 June 2023	829	[66]
Dostarlimab, bintrafusp alfa, cobolimab, feladilimab, GSK 3174998, pembrolizumab	Phase I	Solid tumors	23 June 2016	20 June 2023	829	[66]
Dostarlimab, niraparib	Phase II	Head and neck SCC	4 November 2020	1 June 2027	23	[71]
Dorstarlimab, paclitaxel, encequidar	Phase I	Breast cancer	1 March 2010	December 2031	4000	[72]
Dostarlimab, niraparib	Phase II	Mesothelium	28 January 2019	31 March 2023	200	[73]
Dostarlimab, bevacizumab, niraparib	Phase II	Ovarian cancer	15 November 2018	31 March 2026	125	[74]
Dostarlimab, niraparib, doxorubicin, paclitaxel, gemcitabine, topotecan, bevacizumab	Phase III	Fallopian tube and ovarian cancer	1 December 2020	1 January 2025	427	[75]
Dostarlimab, belantamab, mafodotin,	Phase I/II	Multiple myeloma	7 October 2019	23 February 2028	464	[76]
Dostarlimab, Cobolimab	Phase II	Melanoma	30 April 2020	October 2027	56	[77]
Dostarlimab, carboplatin, paclitaxel	Phase III	Endometrial cancer	18 July 2019	23 December 2026	785	[78]

## Data Availability

Not applicable.
